# The link between different infection forms of *Porphyromonas gingivalis* and acute myocardial infarction: a cross-sectional study

**DOI:** 10.1186/s12903-023-02781-x

**Published:** 2023-02-02

**Authors:** Yingle Wu, Yanyu Wang, Laijing Du, Ke Wang, Shaoxin Wang, Guangping Li

**Affiliations:** 1 Department of Cardiology, The First Affiliated Hospital, and College of Clinical Medicine of Henan University of Science and Technology, Luoyang, 471003 China; 2 Tianjin Key Laboratory of Ionic-Molecular Function of Cardiovascular Disease, Department of Cardiology, Tianjin Institute of Cardiology, The Second Hospital of Tianjin Medical University, Tianjin, 300211 China

**Keywords:** Acute myocardial infarction, Atherosclerosis, Inflammation, Periodontitis, *Porphyromonas gingivalis*

## Abstract

**Background:**

*Porphyromonas gingivalis* (*Pg*) is one of the keystone pathogens involved in periodontitis. The present study aimed to observe the relationship among different infection forms of *Pg*, systemic inflammation, and acute myocardial infarction (AMI).

**Methods:**

A total of 382 patients diagnosed with AMI and 78 patients without coronary heart disease (CHD) were included in the study. DNA from exfoliated oral cells, circulating cell-free DNA (cfDNA), and genomic DNA (gDNA) from blood samples were extracted. The qPCR method was employed to detect *Pg* infection. Clinical characteristics, inflammatory parameters, and severity of coronary artery lesions of the patients were analyzed and compared.

**Results:**

Both the oral colonization and distant invasion of *Pg* correlated positively with systemic inflammation. Multivariate logistic regression analysis suggested that *Pg* positivity in gDNA was correlated with the risk of AMI [Model 1 (odds ratio (OR) = 1.917, 95% confidence interval (CI) 1.108–3.315), Model 2 (OR = 1.863, 95% CI 1.064–3.262), and Model 3 (OR = 1.853, 95% CI 1.042–3.295); *p* < 0.05]. *Pg* positivity in cfDNA and gDNA was related to the severity of coronary artery lesions (cfDNA-positive cases, adjusted OR = 1.577, *p* < 0.05; gDNA-positive cases, adjusted OR = 1.976, *p* < 0.01).

**Conclusions:**

The distant invasion and colonization of *Pg* were the risk factors of AMI. They also affected the severity of CHD, indicating that periodontitis severity and distant invasion of periodontal pathogens were related to CHD. The presence of *Pg* was likely able to drive systemic inflammation, suggesting that there was an inflammatory relationship between periodontitis and AMI.

## Background

Cardiovascular disease (CVD) is the leading cause of death worldwide [1]. Atherosclerosis is the pathological basis of CVD. In addition to traditional risk factors, chronic inflammatory process is one of the important CVD mechanisms [2]. Endothelial dysfunction caused by immune and inflammatory responses is the earliest and most significant process in atherosclerosis [3]. Therefore, chronic infectious diseases, such as periodontitis (PD), have recently become considered responsible for CVD [4–6]. In 1993, DeStefano et al. [7] discovered that PD is one of the risk factors for coronary heart disease (CHD). Since then, the impact of PD on CVD has become the focus of research studies. A growing number of studies have reported a positive correlation between PD and CVD [5]. Several clinical studies have observed the relationship between CVD and oral examination data for patients with PD, such as the number of retained teeth, bleeding on probing, periodontal pocket depth, etc. [8, 9]. However, the American Heart Association [4] has noted that previous data have been inconclusive regarding whether the relationship between PD and CVD is causal or coincidental. Therefore, it is in need of further evaluation.

*Porphyromonas gingivalis* (*Pg*) is the most important pathogen of PD. Based on animal experiments, it has been confirmed that *Pg* is closely related to the initiation and development of many systemic diseases, such as atherosclerosis, cancer, and Alzheimer’s disease [10–12]. Nevertheless, few clinical studies have been conducted to directly observe the effect of pathogens that cause PD in patients with CVD.

Thus, the purpose of the present study was to investigate the presence of *Pg* DNA in the oral cavity and blood samples of patients with acute myocardial infarction (AMI) and non-CHD patients, and analyze its relationship with the incidence of AMI, inflammatory markers, and severity of coronary artery disease, with the aim to optimize early risk stratification for CHD patients and guide clinical treatment.

## Methods

### Study population

**Fig. 1:**
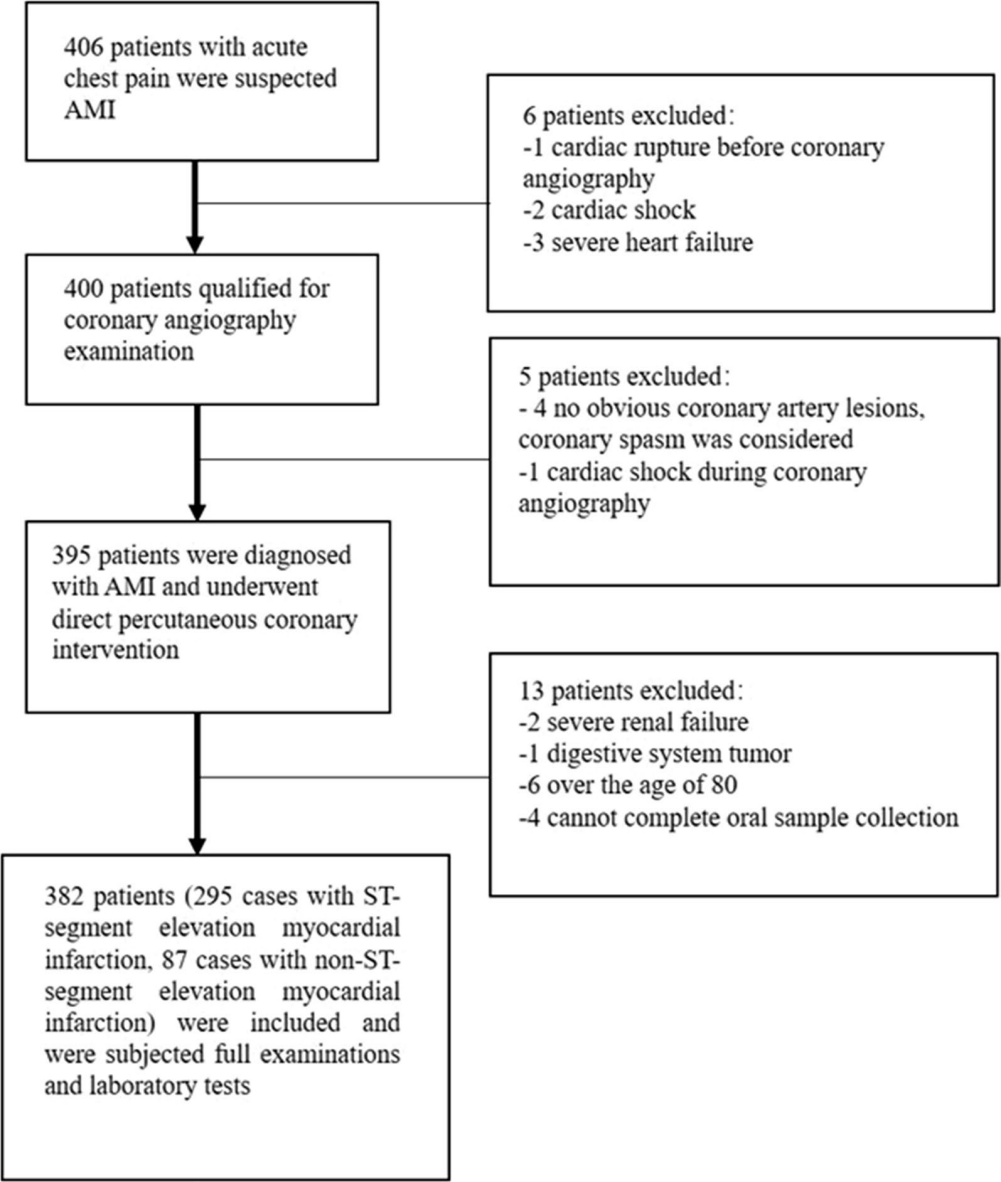
The flow chart for enrollment in the case group

**Fig. 2:**
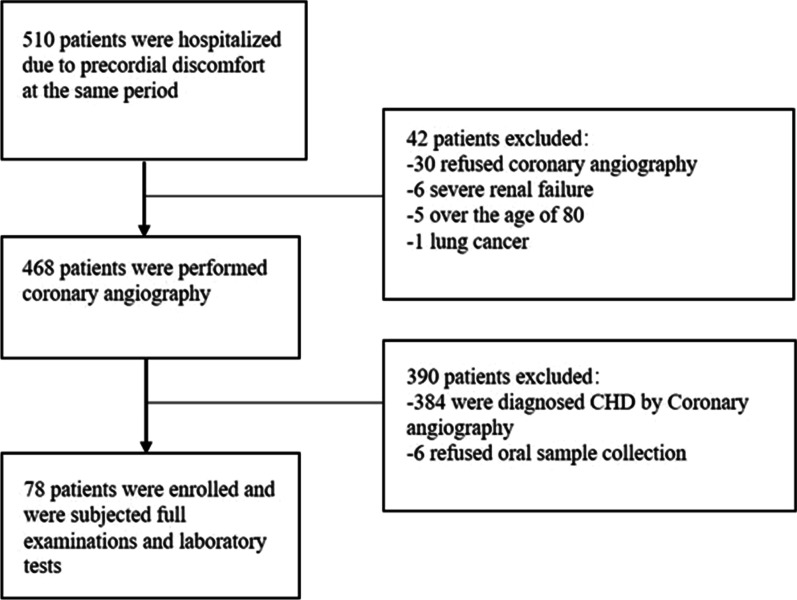
The flow chart for enrollment in the control group

In the present study, the inclusion criteria for the case group were patients diagnosed with AMI, those who underwent direct percutaneous coronary intervention, and individuals < 75 years of age (Fig. 1). The control group consisted of 78 patients < 75 years of age who were hospitalized due to precordial discomfort during the same period. In addition, coronary angiography was performed to exclude CHD (Fig. 2). The patients were fully informed about the study and participated voluntarily. Diagnostic criteria for AMI conformed to the present AMI guidelines [13]. Exclusion criteria included severe heart failure symptoms (NYHA III or Killip II or above); patients with other serious systemic diseases, such as malignant tumors and rheumatic immune system diseases, that affect life expectancy; patients with severe renal failure [serum creatinine > 2.0 mg/dL (176.8 μmol/L)], those undergoing hemodialysis, or individuals suffering from severe liver diseases before the operation; patients with poor compliance judged by the researcher or patients who could not complete the study as required; patients with severe cognitive dysfunction or those unable to communicate for other reasons.

The present study was approved by the ethical review committee of the First Affiliated Hospital of Henan University of Science and Technology, Luoyang, China (approval ID: 2022-03-B029). All patients included in the study were fully informed about the investigation and provided written consent of participation. The study protocol conformed to the ethical guidelines of the 1975 Declaration of Helsinki.

### Clinical data collection

Complete patient medical record information was collected for all participants. All patients underwent coronary angiography, and all angiography procedures were performed using a Philips coronary angiography device (Integris BH, 5000; Philips, Netherlands). Selective coronary angiography was carried out utilizing the radial artery approach with a 6F catheter. Two independent operators determined the percentage of coronary artery stenosis. A syntax scoring system was used to evaluate the severity of coronary artery lesions.

### Quantification of *Pg* DNA in the oral cavity and circulatory system

Prior to performing coronary angiography, oral samples were collected from the gingival tissues of the second and third molars in the four quadrants of the subjects’ oral cavities using disposable oral flocked swabs. Blood samples were obtained from the radial artery through a 6.0F sheath after radial artery puncture.

Oral samples were centrifuged at 12,000 rpm at room temperature and the supernatant was discarded. After adding 50 µL of Tris–EDTA (10-mM Tris, 1-M EDTA, pH 8.0) to the precipitate, it was resuspended in preparation for qPCR. Blood samples were centrifuged at 2500 rpm for 10 min to separate the serum and buffy coat. Circulating cell-free DNA (cfDNA) and genomic DNA (gDNA) were extracted from blood samples for qPCR according to the instructions for the kits (DP348-02, DP339; Tiangen Biotech Co., Ltd., Beijing, China).

The positive rate of *Pg* was determined using qPCR and specific primer probe sequences were as Kuboniwa et al. [14] reported. The designed primer sequences were as follows: *P. gingivalis* forward, 5′-ACCTTACCCGGGATTGAAATG-3′, *P. gingivalis* reverse, 5′-CAACCATGCAGCACCTACATA-GAA-3′; *P. gingivalis* probe, 5′-FAMATGACTGATGGTGAAAA-CCGTCTTCCCTTC-TARMA-3′. Both the primer and probe sequences were synthesized by GENEWIZ Biotechnology Co., Ltd, Suzhou. The PCR system consisted of the following: 10 μL of Ace qPCR Probe Master Mix (Q112-03; Vazyme Biotech Co., Ltd., Nanjing, China), 10-μmol forward (0.5 μL) and reverse (0.5 μL) primers, 0.2-pmoL TaqMan probe, and 2 μL of sample including 50 ng of DNA. DEPC water was added to achieve a total volume of 20 μL. The above system solution was added into the PCR strip tubes. The amplification was performed in the BioRad CFX96TM real-time PCR system at 95ºC for 10 min for a total of 40 PCR cycles (95 °C, 10 s; 60 °C, 60 s). The amplification results were analyzed using the CFX Maestro™ software.

### Statistical analysis

Measurement data were expressed as means and standard deviations ($\overline{x} \pm s$). The differences between groups were compared using a *t*-test if the data satisfied the normal distribution and homogeneity test of variance requirements. Wilcoxon rank sum test was utilized if the data did not satisfy the normal distribution or homogeneity test of variance requirements. Count data were expressed using frequency (percentage), and the differences between the groups were compared by the Pearson’s χ^2^ test. Spearman’s rank analysis was carried out to identify the association between *Pg* and systemic inflammatory factors. The differences in inflammatory parameters of different infection forms of *Pg* in AMI patients were compared with a one-way analysis of variance. Multivariate logistic regression analysis was performed to evaluate the relationship among the different infection forms of *Pg*, AMI, and severity of coronary artery lesions. SPSS statistical software for Windows, version 22.0 (SPSS, Chicago, IL, USA) was employed to sort and analyze the data. All statistical tests were bilateral, with *p* < 0.05 considered to indicate statistical significance.

## Results

### Basic clinical data

A total of 382 patients with a mean age of 57.51 ± 9.92 years diagnosed with AMI (295 cases with ST-segment elevation myocardial infarction, 87 cases with non-ST-segment elevation myocardial infarction) were included in the case group. Then, 78 non-CHD patients with a mean age of 56.85 ± 8.96 years and confirmed non-CHD status using coronary angiography were included in the control group. There were statistically significant differences (*p* < 0.05, Table 1) in proportion of male patients [275 (72%) vs. 47 (60%)], previous history of CHD [54 (14%) vs. 0 (0%)], diabetes status [152 (40%) vs. 20 (26%)], and current smoking status [104 (27%) vs. 12 (15%)] in the AMI group compared to the control group. Indices, such as total cholesterol, low-density lipoprotein, high-density lipoprotein, and inflammatory indexes, represented statistical differences between the non-CHD group and AMI group (Table 1). Most AMI patients were directly transferred to the interventional operating room. Therefore, the clinical data in the present study did not include information such as height and weight.

**Table 1:** Comparison of baseline characteristics between the AMI group and the control group

Indexes	AMI group (*n* = 382)	Control group (*n* = 78)	*p* value
*Demographic characteristics*			
Gender [N (%)]			
Male	275 (72)	47 (60)	< 0.05
Female	107 (28)	31 (40)	
Age [years]	57.51 ± 9.92	61.14 ± 10.19	NS
Medical history [N (%)]			
Hypertension	258 (68)	45 (58)	NS
Diabetes	152 (40)	20 (26)	< 0.05
CHD [N (%)]	54 (14)	0 (0)	< 0.01
Current smoking	104 (27)	12 (15)	< 0.05
Previous medication [N (%)]			
Aspirin	382 (100)	5 (6)	< 0.01
Clopidogrel/Ticagrelor	382 (100)	0 (0)	< 0.01
Statins	375 (98)	15 (19)	< 0.01
β-blocker	288 (75)	40 (51)	< 0.01
ACEIs/ARBs	249 (65)	37 (47)	< 0.01
Laboratory indexes			
WCC [× 10^9^·L^−1^]	7.63 ± 2.29	6.49 ± 1.98	< 0.01
NEUT [× 10^9^·L^−1^]	5.55 ± 244	4.59 ± 2.09	< 0.01
Hb [g·L^−1^]	125.72 ± 14.16	124.31 ± 13.45	NS
CK [U·L^−1^]	335.90 ± 432.62	78.32 ± 15.15	< 0.01
CKMB [U·L^−1^]	28.58 ± 33.57	10.29 ± 5.87	< 0.01
Hs-cTnI [ng·mL^−1^]	13.05 ± 7.31	0.02 ± 0.01	< 0.01
ALT [U·L^−1^]	27.20 ± 13.08	26.01 ± 11.23	NS
AST [U·L^−1^]	31.13 ± 11.30	28.55 ± 8.04	NS
TBIL [μmol·L^−1^]	13.05 ± 3.94	13.82 ± 4.24	NS
DBIL [μmol·L^−1^]	3.64 ± 1.99	3.60 ± 2.17	NS
IBIL [μmol·L^−1^]	9.41 ± 3.97	9.67 ± 3.84	NS
TCHO [mmol·L^−1^]	4.24 ± 0.88	3.93 ± 0.84	< 0.01
HDL-C [mmol·L^−1^]	1.09 ± 0.20	1.13 ± 0.18	NS
LDL-C [mmol·L^−1^]	2.82 ± 0.73	2.60 ± 0.68	< 0.05
TRIG [mmol·L^−1^]	1.41 ± 0.33	1.30 ± 0.38	< 0.05
Lpa [mg·L^−1^]	201.31 ± 105.15	177.20 ± 97.08	NS
CR [μmol·L^−1^]	80.73 ± 13.73	77.70 ± 13.31	NS
UA [μmol·L^−1^]	342.06 ± 87.74	334.49 ± 88.51	NS
HCY [μmol·L^−1^]	15.64 ± 9.45	14.67 ± 7.14	NS
Inflammatory indicators			
FIB [g·L^−1^]	3.07 ± 0.60	2.90 ± 0.59	< 0.05
hsCRP [mg·L^−1^]	5.06 ± 2.31	4.40 ± 2.64	< 0.05
ESR [mm·h^−1^]	7.93 ± 3.57	5.78 ± 3.12	< 0.01
PCT [ng·mL^−1^]	0.33 ± 0.17	0.26 ± 0.12	< 0.01
Cardiac ultrasound results			
LAD [mm]	37.86 ± 5.50	38.22 ± 4.79	NS
LVEDD [mm]	48.14 ± 7.63	47.99 ± 6.97	NS
LVEF [%]	53.36 ± 4.50	57.94 ± 4.42	< 0.01
*Pg* (+) [N (%)]			
Oral cavity	209 (55)	34 (44)	NS
cfDNA	177 (46)	26 (33)	< 0.05
gDNA	152 (40)	20 (26)	< 0.05

*NS* not significant, *CHD* coronary heart disease, *ACEIs/ARBs* angiotensin-converting enzyme inhibitors/angiotensin receptor blockers, *WCC* white cell count, *NEUT* neutrophil count, *Hb* hemoglobin, *CK* creatine kinase, *CKMB* myocardial bound creatine kinase, *hs-cTnI* hypersensitive cardiac troponin I, *ALT* alanine transferase, *AST* aspartate aminotransferase, *TBIL* total bilirubin, *DBIL* direct bilirubin, *IBIL* indirect bilirubin, *TCHO* total cholesterol, *HDL-C* high-density lipoprotein cholesterol, *LDL-C* low-density lipoprotein cholesterol, *TRIG* triglyceride, *Lpa* lipoprotein a, *CR* creatinine, *UA* uric acid, *HCY* homocysteine, *FIB* fibrinogen, *hsCRP* high sensitivity C-reactive protein, *ESR* erythrocyte sedimentation rate, *PCT* procalcitonin, *LAD* left atrial diameter, *LVEDD* left ventricular end-diastolic dimension, *LVEF* left ventricular ejection fraction, *Pg* *Porphyromonas gingivalis*, *cfDNA* circulating free DNA, *gDNA* genomic DNA

### Comparison of *Pg* DNA positivity

There was no significant difference in *Pg* DNA positivity in the oral cavity between the AMI and non-CHD groups [209 cases (55%) vs. 34 cases (44%), *p* > 0.05]. The positive rate of *Pg* in the blood samples was statistically different between the two groups [cfDNA-positive in 177 cases (46%) vs. 26 cases (33%), *p* < 0.05; gDNA-positive in 152 cases (40%) vs. 20 cases [26%], *p* < 0.05; Table 1].

### Association between different infection forms of *Pg* and systemic inflammation

**Fig. 3:**
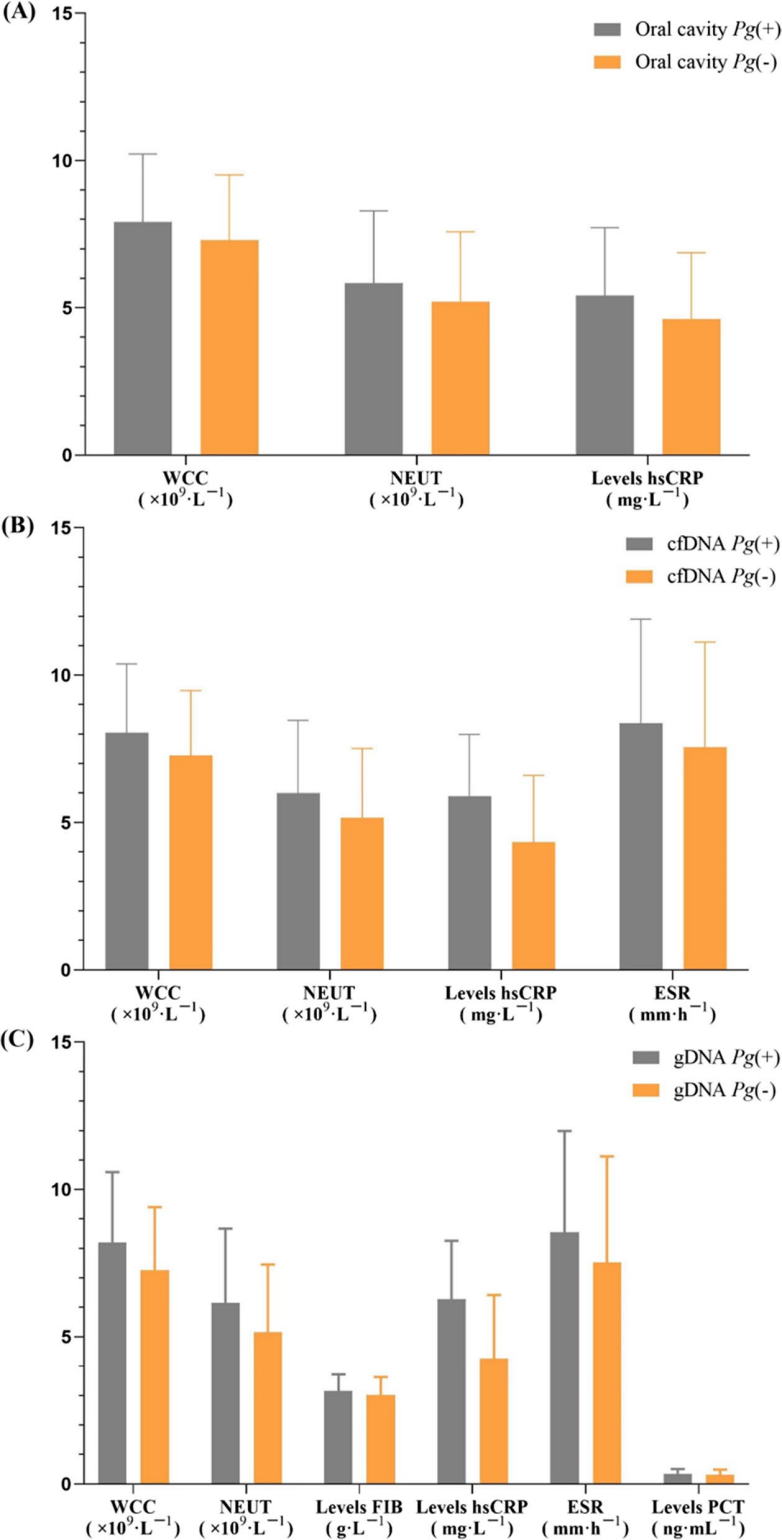
Effect of *Pg* on inflammatory indexes of AMI group. **A** Showed that WCC, NEUT, and hsCRP levels were higher in patients positive for *Pg* in the oral cavity, *p* < 0.05. **B** Showed that WCC, NEUT, hsCRP, and ESR levels were higher in patients positive for *Pg* in cfDNA,* p* < 0.05. **C** Showed that WCC, NEUT, FIB, hsCRP, ESR and PCT levels were higher in patients positive for *Pg* in gDNA, *p* < 0.05. *WCC* white cell count, *NEUT* neutrophil count, *hsCRP* high sensitivity C-reactive protein, *ESR* erythrocyte sedimentation rate, *FIB* fibrinogen, *PCT* procalcitonin

Spearman’s rank correlation analysis was performed to identify the association between different infection forms of *Pg* and systemic inflammatory factors (Table 2). In addition, patients in the AMI group were divided into groups based on the presence of *Pg*. The inflammatory indexes in AMI patients with *Pg* infection were significantly high (Fig. 3), especially in patients with *Pg* positivity in gDNA (Table 3)*.*

**Table 2:** Spearman’s rank correlation for different infection forms of *Pg* with the values of inflammatory parameters

Infammatory parameters	*Pg* (+)	Correlations	
		R	*p*
WCC	Oral cavity	0.120	0.010
	cfDNA	0.147	0.002
	gDNA	0.186	< 0.001
NEUT	Oral cavity	0.100	0.032
	cfDNA	0.136	0.003
	gDNA	0.166	< 0.001
FIB	Oral cavity	0.131	0.005
	cfDNA	0.111	0.017
	gDNA	0.121	0.009
hsCRP	Oral cavity	0.132	0.005
	cfDNA	0.298	< 0.001
	gDNA	0.412	< 0.001
ESR	Oral cavity	0.074	0.114
	cfDNA	0.112	0.017
	gDNA	0.135	0.004
PCT	Oral cavity	0.089	0.056
	cfDNA	0.062	0.183
	gDNA	0.108	0.020

*WCC* white cell count, *NEUT* neutrophil count, *FIB* fibrinogen, *hsCRP* high sensitivity C-reactive protein, *ESR* erythrocyte sedimentation rate, *PCT* procalcitonin, *Pg* *Porphyromonas gingivalis*, *cfDNA* circulating cell-free DNA, *gDNA* genomic DNA

**Table 3:** The effect of different infection forms of *Pg* on the values of inflammatory parameters in AMI patients

Infammatory parameters	Oral cavity *Pg* (+)	cfDNA *Pg* (+)	gDNA *Pg* (+)	*p*
WCC	7.15 ± 2.08	7.12 ± 1.68	8.19 ± 2.39	0.013
NEUT	4.96 ± 2.20	5.02 ± 1.94	6.15 ± 2.52	0.009
FIB	3.15 ± 0.62	2.90 ± 0.73	3.16 ± 0.56	0.238
hsCRP	2.79 ± 1.54	3.60 ± 0.89	6.27 ± 1.98	< 0.001
ESR	7.25 ± 3.67	7.28 ± 3.92	8.55 ± 3.44	0.067
PCT	0.32 ± 0.12	0.29 ± 0.28	0.35 ± 0.16	0.372

*WCC* white cell count, *NEUT* neutrophil count, *FIB* fibrinogen, *hsCRP* high sensitivity C-reactive protein, *ESR* erythrocyte sedimentation rate, *PCT* procalcitonin, *Pg* *Porphyromonas gingivalis*, *cfDNA* circulating cell-free DNA, *gDNA* genomic DNA

### Association between *Pg* infection and AMI

Univariate and multivariable logistic regression were performed with the occurrence of AMI (No: 0; Yes: 1) was used as the dependent variable, and *Pg* positivity was used as the variable to be adjusted first. The results showed that *Pg* positivity in gDNA was an independent risk factor for AMI [Model 1 (odds ratio (OR) = 1.917, 95% confidence interval (CI) 1.108–3.315), Model 2 (OR = 1.863, 95% CI 1.064–3.262), and Model 3 (OR = 1.853, 95% CI 1.042–3.295) (*p* < 0.05); Table 4].

**Table 4:** Correlation between *Pg* infection and risk of AMI occurrence

*Pg* (+)	Case number (N)	AMI (N)	Model 1^a^		Model 2^b^		Model 3^c^	
			OR (95% CI)	*p* value	OR (95% CI)	*p* value	OR (95% CI)	*p* value
Oral cavity	243	209	1.563 (0.957–2.554)	0.74	1.513 (0.900–2.543)	0.12	1.457 (0.853–2.486)	0.17
cfDNA	203	177	1.727 (1.035–2.881)	< 0.05	1.711 (1.005–2.911)	< 0.05	1.671 (0.967–2.890)	0.07
gDNA	172	152	1.917 (1.108–3.315)	< 0.05	1.863 (1.064–3.262)	< 0.05	1.853 (1.042–3.295)	< 0.05

*Pg* *Porphyromonas gingivalis*, *cfDNA* circulating free DNA, *gDNA* genomic DNA

^a^Variables without adjustment

^b^Adjusting gender, age, and medical history (hypertension, diabetes, and current smoking)

^c^Adjusting the variables in the model^b^ as well as the low-density lipoprotein, high-density lipoprotein cholesterol, triglyceride, lipoprotein a, and homocysteine

### Association between *Pg* infection and severity of coronary artery lesions

The severity of coronary artery lesions in AMI patients was divided into a mild (Syntax score < 23) and a moderate-severe (Syntax score ≥ 23) groups. In univariate logistic regression analysis, the results indicated that *Pg* positivity in the circulatory system was related to the severity of coronary artery. The same result was obtained when the confounding factors were adjusted (Table 5).

**Table 5:** Correlation between *Pg* and severity of coronary artery lesions in AMI patients

*Pg* (+)	Model 1^a^	*p* value	Model 2^b^	*p* value	Model 3^c^	*p* value
	OR (95% CI)		OR (95% CI)		OR (95%CI)	
Oral cavity	1.501 (0.992–2.271)	0.06	1.463 (0.950–2.251)	0.08	1.427 (0.923–2.207)	0.11
cfDNA	1.631 (1.081–2.462)	< 0.05	1.615 (1.060–2.460)	< 0.05	1.577 (1.030–2.412)	< 0.05
gDNA	2.001 (1.316–3.042)	< 0.01	1.995 (1.307–3.047)	< 0.01	1.976 (1.287–3.033)	< 0.01

*Pg* *Porphyromonas gingivalis*, *cfDNA* circulating free DNA, *gDNA* genomic DNA

^a^Variables without adjustment

^b^Adjusting gender, age, and medical history (hypertension, diabetes, and current smoking)

^c^Adjusting the variables in the model^b^ as well as the low-density lipoprotein, high-density lipoprotein cholesterol, triglyceride, lipoprotein a, and homocysteine

## Discussion

The present study for the first time evaluated the different infection forms of *Pg* by detecting the presence of *Pg* DNA using the qPCR method and analyzed its relationship with AMI. The main study findings were as follows: (1) *Pg* correlated positively with systemic inflammation regardless of the *Pg* infection mode, and systemic inflammatory molecular levels increased significantly; (2) *Pg* positivity in the circulatory system (cfDNA and gDNA) was an independent risk factor for first-time AMI and was related to the severity of coronary artery, with a possible clinical significance for optimizing risk stratification in CHD patients; and (3) the study findings further strengthened the possibility of an independent relationship between PD and CVD manifestations.

The present study supported the idea that the main mechanism for PD affecting the development of atherosclerosis was the direct invasion of endothelial cells by periodontal pathogens [6]. Furthermore, cfDNA consisted of extracellular DNA fragments present in the serum that may be derived from both normal and diseased cells. *Pg* positivity in cfDNA indicated that *Pg* invaded the circulatory system. In addition, *Pg* positivity in gDNA indicated that *Pg* colonized the circulatory system with persistent presence in the cells, further resulting in a lasting inflammatory state. The study results also showed a positive association between gDNA *Pg* positivity and AMI, which remained following adjustment for the differences in clinical characteristics between patients and controls. This reinforces the possibility of an independent relationship between PD and the risk for CVD presently expressed as AMI, which depends on whether the distant invasion and colonization of endothelial cells by periodontal pathogens were involved.

On one hand, *Pg* can leave oral epithelial cells via the endocytic recycling pathway and infect other cells or enter the circulatory system [15]. Live *Pg* can be detected in human aortic endothelial cells [16], human pancreatic tumor cells [17], and human myeloid dendritic cells [18]. *Pg* DNA can be detected in atherosclerotic plaques [19]. Repeated intravenous injection of *Pg* can aggravate the progression of atherosclerosis in mice, and the size of aortic lesions inoculated with *Pg* was twice as large as that of the control group [10]. On the other hand, Dietrich et al. [8] have found that patients with severe PD had an increased risk of the first coronary artery event compared to patients without PD or those with mild PD. In the PAROKRANK study [9] has found that the risk of the first AMI increased significantly in patients with moderate to severe PD. It has been speculated that this was because patients with severe PD were more likely to transfer microorganisms from dental pockets into the bloodstream by chewing and via dental treatments causing bacteremia and systemic inflammation [20]. Another finding of the present study illustrated that *Pg* positivity in cfDNA and gDNA samples from AMI patients was positively correlated with the severity of coronary artery disease (Table 5), which again demonstrated that direct invasion of periodontal pathogens was related to the development of atherosclerosis. Such studies indicate that *Pg* can enter the circulatory system and directly act on the lesion site to promote the development of atherosclerosis. So far, it has not been determined how the bacteria existing in cells influence atherosclerosis. However, in vitro experiments have shown that *Pg* could trigger the formation of foam cells or result in their persistent presence in the cells, causing a secondary inflammatory state and leading to endothelial dysfunction [21]. In addition, it inhibits cell apoptosis [22], suggesting the inflammatory relationship between *Pg* and atherosclerosis, which is consistent with the present study results.

The present study showed that *Pg* correlated positively with systemic inflammation regardless of the *Pg* infection mode (Table 2). The same results were observed in patients with AMI (Fig. 3), especially in individuals with *Pg* positivity in gDNA (Table 3). These results supported another hypothesis that periodontal pathogens affect the occurrence and development of atherosclerosis, increasing systemic inflammatory molecular levels through indirect pathways.

CHD is an inflammatory disease, and inflammation plays an important role in the development and manifestations of CHD [23]. Determining the levels of inflammatory markers may be important for assessing the risk of CHD [24–26]. PD can stimulate a systemic inflammatory response, resulting in a long-term increase in the levels of different cytokines, which are also related to atherosclerotic vascular diseases [27]. However, most previous studies have focused on the relationship between periodontal parameters, inflammatory factors, and CVD [28, 29]. In the present study, the effects of different infection forms of the PD pathogen *Pg* on systemic inflammatory factors were directly observed in the human body. It was discovered that *Pg* could increase systemic inflammatory factors whether it was colonized in the oral cavity or invaded a distant area. At the same time, patients with *Pg*-positive gDNA and AMI had more severe systemic inflammatory reactions, further supporting the direct effect of periodontal pathogens.

In summary, *Pg* had an independent influence on the occurrence of AMI and the severity of CHD. It could affect the development of CHD through direct invasion and triggering systemic inflammatory reactions. Although there is insufficient evidence to clarify the potential benefits of periodontal treatment for secondary prevention of CVD [5, 30], PD is widespread, and some studies suggest that it may be a changeable risk factor for CVD. Therefore, we should pay attention to the periodontal health of patients with CVD and take appropriate preventive and therapeutic measures. In fact, when clinicians are faced with patients with AMI, it is difficult to obtain detailed periodontal test data for patients at the first time, such as the number of retained teeth, bleeding on probing, and so on. However, determining whether patients are infected with related pathogenic bacteria and the level of related inflammatory mediators may play a guiding role in further clinical interventions.

There were some limitations in this study. First, this was a cross-sectional, single-center study with relatively small sample size. Therefore, it could not determine the causality between *Pg* and AMI. Second, other inflammatory processes might coexist in AMI patients. Third, due to the sample size, the effect of *Pg* on inflammatory molecular levels in non-CHD patients was not investigated. Finally, the present study results showed no significant difference in *Pg* DNA positivity in the oral cavity between the two groups [209 cases (55%) vs. 34 cases (44%), *p* > 0.05], which contradicted previous study results [31, 32]. This may be explained by the use of a specific and possibly less efficient oral sampling method. In addition, periodontal diagnosis was not performed in the study. Therefore, there may be some bias in the results and further study is needed.

## Conclusion

PD pathogen invasion and colonization in the circulatory system was one of the risk factors of first-time AMI and was related to the severity of coronary artery lesions. The systemic inflammation was more evident in AMI patients with *Pg* positivity in the circulatory system, which may suggest a potential inflammatory link between PD and AMI. Two conclusions can be drawn from the present study. First, *Pg* positivity in the circulatory system was significantly and positively correlated with AMI. Second, *Pg* promoted systemic inflammation response. Future multicenter, prospective, and randomized clinical trials are necessary to determine whether the treatment of PD and removal of its pathogenic bacteria can help prevent the occurrence or recurrence of CVD.

## Data Availability

The datasets used and/or analyzed during the current study are available from the corresponding author on reasonable request.

## Abbreviations

*Pg*: *Porphyromonas gingivalis*
cfDNA: Circulating free DNA
gDNA: Genomic DNA
CHD: Coronary heart disease
AMI: Acute myocardial infarction
CVD: Cardiovascular disease
PD: Periodontitis
